# Supplementary material for: Clinicopathological Factors Related to Recurrence Patterns of Resected Non-Small Cell Lung Cancer
Source: J Clin Med. 2020 Aug 1;9(8):2473. doi: 10.3390/jcm9082473 (PMC7465649; doi:10.3390/jcm9082473)
Supplement: Supplementary file 1 [file jcm-09-02473-s001.pdf]

Supplementary Materials:

Table 1. Relationship between clinicopathologic factors and organ-specific recurrence (A, mediastinal lymph node; B, lung; C, brain; D, bone; E, liver; and F, adrenal gland) on chi-squared/Fisher test.

(A)

		**Mediastinal Lymph Node Metastasis**											
		**All Cases**						**Recurrence Cases**					
		**Initial Recurrence**			**Final Recurrence**			**Initial Recurrence**			**Final Recurrence**		
**Factors**		**Present (%)**	**Absent (%)**	***p***	**Present (%)**	**Absent (%)**	***p***	**Present (%)**	**Absent (%)**	***p***	**Present (%)**	**Absent (%)**	***p***
Sex	male	52 (13)	356 (87)	0.001	55 (13)	353 (87)	0.022	52 (36)	93 (64)	0.030	55 (38)	90 (62)	0.145
	female	17 (6)	263 (93)		22 (8)	258 (92)		17 (22)	61 (78)		22 (28)	56 (72)	
Age	≥70	35 (9)	347 (91)	0.398	39 (10)	343 (90)	0.361	35 (32)	75 (68)	0.780	39 (35)	71 (65)	0.774
	<70	34 (11)	272 (89)		38 (12)	268 (88)		34 (30)	79 (70)		38 (34)	75 (66)	
Smoking	ever	46 (10)	436 (90)	0.517	51 (11)	431 (89)	0.437	46 (28)	118 (72)	0.119	51 (34)	113 (69)	0.072
	never	23 (11)	183 (89)		26 (13)	180 (87)		23 (39)	36 (61)		26 (44)	33 (56)	
Pathology	AD	45 (10)	425 (90)	0.560	53 (11)	417 (89)	0.918	45 (29)	109 (71)	0.406	53 (34)	101 (66)	0.958
	others	24 (11)	194 (89)		24 (11)	194 (89)		24 (35)	45 (65)		24 (35)	45 (65)	
Lymphatic permeation	present	45 (15)	262 (85)	0.001	51 (17)	256 (83)	0.001	45 (38)	75 (63)	0.022	51 (43)	69 (58)	0.009
	absent	24 (6)	357 (94)		26 (7)	355 (93)		24 (23)	79 (77)		26 (25)	77 (75)	
Vascular invasion	present	56 (15)	330 (85)	0.001	63 (16)	323 (84)	0.001	56 (32)	119 (68)	0.514	63 (36)	112 (64)	0.378
	absent	13 (4)	289 (96)		14 (5)	288 (95)		13 (27)	35 (73)		14 (29)	34 (71)	
Pleural invasion	present	36 (25)	107 (75)	0.001	41 (29)	102 (71)	0.001	36 (28)	94 (72)	0.215	41 (32)	89 (68)	0.267
	absent	33 (6)	512 (94)		36 (7)	509 (93)		33 (35)	60 (65)		36 (39)	57 (61)	
Pulmonary metastasis	present	4 (10)	37 (90)	0.952	4 (10)	37 (90)	0.764	4 (22)	14 (78)	0.404	4 (22)	14 (78)	0.252
	absent	65 (10)	582 (90)		73 (11)	574 (89(		65 (32)	140 (68)		73 (36)	132 (64)	
Invasive size	>3 cm	45 (16)	234 (84)	0.001	49 (18)	230 (82)	0.001	45 (33)	93 (67)	0.493	49 (36)	89 (67)	0.695
	≤3 cm	24 (6)	385 (94)		28 (7)	381 (93)		24 (28)	61 (72)		28 (33)	57 (67)	
Lymph node metastasis	present	48 (25)	144 (75)	0.001	51 (27)	141 (73)	0.001	48 (41)	68 (59)	0.001	51 (44)	65 (56)	0.002
	absent	21 (4)	475 (96)		26 (5)	470 (95)		21 (20)	86 (80)		26 (24)	81 (76)	
Adjuvant therapy	performed	28 (16)	142 (84)	0.001	30 (18)	140 (82)	0.002	28 (32)	60 (68)	0.819	30 (34)	58 (66)	0.912
	undone	41 (8)	477 (92)		47 (9)	471 (91)		41 (30)	94 (70)		47 (35)	88 (65)	

Pathological stage	II–III	57 (6)	252 (94)	0.001	61 (20)	248 (80)	0.001	57 (36)	103 (64)	0.016	61 (38)	99 (62)	0.072
	I	12 (3)	367 (97)		16 (4)	363 (96)		12 (19)	51 (81)		16 (25)	47 (75)	

(B)

		Lung Metastasis											
		All Cases						Recurrence Cases					
		Initial Recurrence			Final Recurrence			Initial Recurrence			Final Recurrence		
**Factors**		**Present (%)**	**Absent (%)**	***p***	**Present (%)**	**Absent (%)**	***p***	**Present (%)**	**Absent (%)**	***p***	**Present (%)**	**Absent (%)**	***p***
Sex	male	47 (12)	361 (88)	0.435	61 (15)	347 (85)	0.438	47 (32)	98 (68)	0.739	61 (42)	84 (58)	0.557
	female	27 (10)	253 (90)		36 (13)	244 (87)		27 (35)	51 (65)		36 (46)	42 (54)	
Age	≥70	35 (9)	347 (91)	0.132	41 (11)	341 (89)	0.005	35 (32)	75 (68)	0.669	41 (37)	69 (63)	0.064
	<70	39 (13)	267 (87)		56 (18)	250 (82)		39 (35)	74 (65)		56 (50)	57 (50)	
Smoking	ever	55 (11)	427 (89)	0.396	74 (15)	408 (85)	0.148	55 (34)	109 (66)	0.852	74 (45)	90 (55)	0.415
	never	19 (9)	187 (91)		23 (11)	183 (89)		19 (32)	40 (68)		23 (39)	36 (61)	
Pathology	AD	54 (11)	416 (89)	0.362	70 (15)	400 (85)	0.379	54 (35)	100 (65)	0.373	70 (45)	84 (55)	0.379
	others	20 (9)	198 (91)		27 (12)	191 (88)		20 (29)	49 (71)		27 (39)	42 (61)	
Lymphatic permeation	present	36 (12)	271 (88)	0.461	51 (17)	256 (83)	0.089	36 (30)	84 (70)	0.276	51 (43)	69 (58)	0.746
	absent	38 (10)	343 (90)		46 (12)	335 (88)		38 (37)	65 (63)		46 (45)	57 (55)	
Vascular invasion	present	52 (13)	334 (87)	0.009	71 (18)	315 (82)	0.001	52 (30)	123 (70)	0.036	71 (41)	104 (59)	0.092
	absent	22 (7)	280 (93)		26 (9)	276 (91)		22 (46)	26 (54)		26 (54)	22 (46)	
Pleural invasion	present	45 (31)	98 (69)	0.001	58 (41)	85 (59)	0.001	45 (35)	85 (65)	0.591	58 (45)	72 (55)	0.691
	absent	29 (5)	516 (95)		39 (7)	506 (93)		29 (31)	64 (69)		39 (42)	54 (58)	
Pulmonary metastasis	present	10 (24)	31 (76)	0.004	12 (29)	29 (81)	0.004	10 (56)	8 (44)	0.036	12 (67)	6 (33)	0.039
	absent	64 (10)	583 (90)		85 (13)	562 (87)		64 (31)	141 (69)		85 (41)	120 (59)	
Invasive size	>3 cm	50 (18)	229 (82)	0.001	62 (22)	217 (78)	0.001	50 (36)	88 (64)	0.218	62 (45)	76 (55)	0.583
	≤3 cm	24 (6)	385 (91)		35 (9)	374 (91)		24 (28)	61 (72)		35 (41)	50 (59)	
Lymph node metastasis	present	33 (17)	159 (83)	0.001	47 (24)	145 (76)	0.001	33 (28)	83 (72)	0.118	47 (41)	69 (59)	0.350
	absent	41 (8)	455 (92)		50 (10)	446 (90)		41 (38)	66 (62)		50 (47)	57 (53)	
Adjuvant therapy	performed	26 (15)	144 (85)	0.028	37 (22)	133 (78)	0.001	26 (30)	62 (70)	0.352	37 (42)	51 (58)	0.724
	undone	48 (9)	470 (91)		60 (12)	458 (88)		48 (36)	87 (64)		60 (44)	75 (56)	
Pathological stage	II–III	54 (17)	255 (83)	0.001	69 (22)	240 (78)	0.001	54 (34)	106 (66)	0.775	69 (43)	91 (57)	0.786

	I	20 (5)	359 (95)		28 (7)	351 (93)		20 (32)	43 (68)		28 (44)	35 (56)	

(C)

		**Brain Metastasis**											
		**All Cases**						**Recurrence Cases**					
		**Initial Recurrence**			**Final Recurrence**			**Initial Recurrence**			**Final Recurrence**		
**Factors**		**Present (%)**	**Absent (%)**	***p***	**Present (%)**	**Absent (%)**	***p***	**Present (%)**	**Absent (%)**	***p***	**Present (%)**	**Absent (%)**	***p***
Sex	male	25 (6)	383 (94)	0.479	46 (11)	362 (89)	0.934	25 (17)	120 (83)	0.088	46 (32)	99 (68)	0.230
	female	21 (8)	259 (93)		31 (11)	249 (89)		21 (27)	57 (73)		31 (40)	47 (60)	
Age	≥70	15 (4)	367 (96)	0.001	28 (7)	354 (93)	0.001	15 (14)	95 (86)	0.011	28 (25)	82 (75)	0.005
	<70	31 (10)	275 (90)		49 (16)	257 (84)		31 (27)	82 (73)		49 (43)	64 (57)	
Smoking	ever	29 (6)	453 (94)	0.282	52 (11)	430 (89)	0.608	29 (18)	135 (82)	0.070	52 (32)	112 (68)	0.140
	never	17 (8)	189 (92)		25 (12)	181 (88)		17 (29)	42 (71)		25 (42)	34 (58)	
Pathology	AD	38 (8)	432 (92)	0.031	67 (14)	403 (86)	0.001	38 (25)	116 (75)	0.026	67 (44)	87 (56)	0.001
	others	8 (4)	210 (96)		10 (5)	208 (95)		8 (12)	61 (88)		10 (14)	59 (86)	
Lymphatic permeation	present	30 (10)	277 (90)	0.004	52 (17)	255 (83)	0.001	30 (25)	90 (75)	0.082	52 (43)	68 (57)	0.003
	absent	16 (4)	365 (96)		25 (7)	356 (93)		16 (16)	87 (84)		25 (24)	78 (76)	
Vascular invasion	present	41 (11)	345 (89)	0.001	67 (17)	319 (83)	0.001	41 (23)	134 (77)	0.048	67 (38)	108 (62)	0.024
	absent	5 (2)	297 (98)		10 (3)	292 (97)		5 (10)	43 (90)		10 (21)	38 (79)	
Pleural invasion	present	24 (17)	119 (83)	0.001	47 (33)	96 (67)	0.001	24 (18)	106 (82)	0.345	47 (36)	83 (64)	0.546
	absent	22 (4)	523 (96)		30 (6)	515 (94)		22 (24)	71 (76)		30 (32)	63 (68)	
Pulmonary metastasis	present	4 (10)	37 (90)	0.417	6 (15)	35 (85)	0.471	4 (22)	14 (78)	0.862	6 (33)	12 (67)	0.911
	absent	42 (6)	605 (94)		71 (11)	576 (89)		42 (20)	163 (80)		71 (35)	134 (65)	
Invasive size	>3 cm	26 (9)	253 (91)	0.022	43 (15)	236 (85)	0.004	26 (19)	112 (81)	0.401	43 (31)	95 (69)	0.177
	≤3 cm	20 (5)	389 (95)		34 (8)	375 (92)		20 (24)	65 (76)		34 (40)	51 (60)	
Lymph node metastasis	present	27 (14)	165 (86)	0.001	46 (24)	146 (76)	0.001	27 (23)	89 (77)	0.309	46 (40)	70 (60)	0.094
	absent	19 (4)	477 (96)		31 (6)	465 (94)		19 (18)	88 (82)		31 (29)	76 (71)	
Adjuvant therapy	performed	23 (14)	147 (86)	0.001	36 (21)	134 (79)	0.001	23 (26)	65 (74)	0.101	36 (41)	52 (59)	0.106
	undone	23 (4)	495 (96)		41 (8)	477 (92)		23 (17)	112 (83)		41 (30)	94 (70)	
Pathological stage	II–III	35 (11)	274 (89)	0.001	59 (19)	250 (81)	0.001	35 (22)	125 (78)	0.463	59 (37)	101 (63)	0.240
	I	11 (3)	368 (97)		18 (5)	361 (95)		11 (17)	52 (83)		18 (29)	45 (71)	

(D)

Factors		Bone Metastasis											
		All Cases						Recurrence Cases					
		Initial Recurrence			Final Recurrence			Initial Recurrence			Final Recurrence		
		Present (%)	Absent (%)	*p*	Present (%)	Absent (%)	*p*	Present (%)	Absent (%)	*p*	Present (%)	Absent (%)	*p*
Sex	male	21 (5)	387 (95)	0.764	37 (9)	371 (91)	0.368	21 (14)	124 (86)	0.665	37 (26)	108 (74)	0.984
	female	13 (5)	267 (95)		20 (7)	260 (93)		13 (17)	65 (83)		20 (26)	58 (74)	
Age	≥70	18 (5)	364 (95)	0.756	27 (7)	355 (93)	0.196	18 (16)	92 (84)	0.647	27 (25)	83 (75)	0.732
	<70	16 (5)	290 (95)		30 (10)	276 (90)		16 (14)	97 (86)		30 (27)	83 (73)	
Smoking	ever	29 (6)	453 (94)	0.047	44 (9)	438 (91)	0.219	29 (18)	135 (82)	0.092	44 (27)	120 (73)	0.469
	never	5 (5)	201 (98)		13 (6)	193 (94)		5 (8)	54 (92)		13 (22)	46 (78)	
Pathology	AD	24 (5)	446 (95)	0.770	43 (9)	427 (91)	0.227	24 (16)	130 (84)	0.834	43 (28)	111 (72)	0.227
	others	10 (5)	208 (95)		14 (6)	204 (94)		10 (14)	59 (86)		14 (20)	55 (80)	
Lymphatic permeation	present	20 (7)	287 (93)	0.088	33 (11)	274 (89)	0.035	20 (17)	100 (83)	0.524	33 (28)	87 (73)	0.474
	absent	14 (4)	367 (96)		24 (6)	357 (94)		14 (14)	89 (86)		24 (23)	79 (77)	
Vascular invasion	present	29 (8)	357 (92)	0.001	46 (12)	340 (88)	0.001	29 (17)	146 (83)	0.293	46 (26)	129 (74)	0.635
	absent	5 (2)	297 (98)		11 (4)	291 (96)		5 (10)	43 (90)		11 (23)	37 (77)	
Pleural invasion	present	21 (15)	122 (85)	0.001	30 (21)	113 (79)	0.001	21 (16)	109 (84)	0.656	30 (23)	100 (77)	0.315
	absent	13 (2)	532 (98)		27 (5)	518 (98)		13 (14)	80 (86)		27 (29)	66 (71)	
Pulmonary metastasis	present	2 (5)	39 (95)	0.984	3 (7)	38 (93)	0.817	2 (11)	16 (89)	0.611	3 (17)	15 (83)	0.367
	absent	32 (5)	615 (95)		54 (8)	593 (92)		32 (16)	173 (84)		54 (26)	151 (74)	
Invasive size	>3 cm	22 (8)	257 (92)	0.003	31 (11)	248 (89)	0.026	22 (16)	116 (84)	0.713	31 (22)	107 (78)	0.177
	≤3 cm	12 (3)	397 (97)		26 (6)	383 (94)		12 (14)	73 (86)		26 (31)	59 (69)	
Lymph node metastasis	present	15 (8)	177 (92)	0.031	29 (15)	163 (85)	0.001	15 (13)	101 (87)	0.317	29 (25)	87 (75)	0.842
	absent	19 (4)	477 (96)		28 (6)	468 (94)		19 (18)	88 (82)		28 (26)	79 (74)	
Adjuvant therapy	performed	10 (6)	160 (94)	0.514	20 (12)	150 (88)	0.058	10 (11)	78 (89)	0.193	20 (23)	68 (77)	0.434
	undone	24 (5)	494 (98)		37 (7)	481 (93)		24 (18)	111 (82)		37 (27)	98 (73)	
Pathological stage	II–III	22 (7)	287 (93)	0.017	37 (12)	272 (88)	0.002	22 (14)	138 (86)	0.322	37 (23)	123 (77)	0.184
	I	12 (3)	367 (97)		20 (5)	359 (95)		12 (19)	51 (81)		20 (32)	43 (68)	

(E)

Liver metastasis

Factors		All cases						Recurrence cases					
		Initial recurrence			Final recurrence			Initial recurrence			Final recurrence		
		Present (%)	Absent (%)	*p*	Present (%)	Absent (%)	*p*	Present (%)	Absent (%)	*p*	Present (%)	Absent (%)	*p*
Sex	male	17 (4)	391 (96)	0.044	19 (5)	389 (95)	0.160	17 (12)	128 (88)	0.108	19 (13)	126 (87)	0.360
	female	4 (1)	276 (99)		7 (3)	273 (98)		4 (5)	74 (95)		7 (9)	71 (91)	
Age	≥70	14 (4)	368 (96)	0.375	14 (4)	368 (96)	0.999	14 (13)	96 (87)	0.095	14 (13)	96 (97)	0.624
	<70	7 (2)	299 (98)		12 (4)	294 (96)		7 (6)	106 (94)		12 (11)	101 (89)	
Smoking	ever	15 (3)	467 (97)	0.999	18 (4)	464 (96)	0.999	15 (9)	149 (91)	0.818	18 (11)	146 (89)	0.596
	never	6 (3)	200 (97)		8 (4)	198 (96)		6 (10)	53 (90)		8 (14)	51 (86)	
Pathology	AD	11 (2)	459 (98)	0.151	16 (3)	454 (97)	0.520	11 (7)	143 (93)	0.082	16 (10)	138 (90)	0.377
	others	10 (5)	208 (95)		10 (5)	208 (95)		10 (14)	59 (86)		10 (14)	59 (86)	
Lymphatic permeation	present	12 (4)	295 (96)	0.270	14 (5)	293 (95)	0.422	12 (10)	108 (90)	0.748	14 (12)	106 (88)	0.997
	absent	9 (2)	372 (98)		12 (3)	369 (97)		9 (9)	94 (91)		12 (12)	91 (88)	
Vascular invasion	present	21 (5)	365 (95)	0.001	24 (6)	362 (94)	0.001	21 (12)	154 (88)	0.012	24 (14)	151 (86)	0.068
	absent	0 (0)	302 (100)		2 (1)	300 (99)		0 (0)	48 (100)		2 (4)	46 (96)	
Pleural invasion	present	12 (8)	131 (92)	0.001	13 (9)	130 (91)	0.001	12 (9)	118 (91)	0.910	13 (10)	117 (90)	0.361
	absent	9 (2)	536 (98)		13 (2)	532 (98)		9 (10)	84 (90)		13 (14)	80 (86)	
Pulmonary metastasis	present	2 (5)	39 (95)	0.360	2 (5)	39 (95)	0.664	2 (11)	16 (89)	0.797	2 (11)	16 (89)	0.940
	absent	19 (3)	628 (97)		24 (4)	623 (97)		19 (9)	186 (91)		24 (12)	181 (88)	
Invasive size	>3 cm	13 (5)	266 (95)	0.068	15 (5)	264 (95)	0.101	13 (9)	125 (91)	0.998	15 (11)	123 (89)	0.640
	≤3 cm	8 (2)	401 (98)		11 (3)	398 (97)		8 (9)	77 (91)		11 (13)	74 (87)	
Lymph node metastasis	present	11 (6)	181 (94)	0.011	13 (7)	179 (93)	0.014	11 (9)	105 (91)	0.972	13 (11)	103 (89)	0.827
	absent	10 (2)	486 (98)		13 (3)	483 (97)		10 (9)	97 (91)		13 (12)	94 (88)	
Adjuvant therapy	performed	6 (4)	164 (96)	0.617	6 (4)	164 (96)	0.999	6 (7)	82 (93)	0.283	6 (7)	82 (93)	0.069
	undone	15 (3)	503 (97)		20 (4)	498 (96)		15 (11)	120 (89)		20 (15)	115 (85)	
Pathological stage	II–III	17 (6)	292 (94)	0.001	19 (6)	290 (94)	0.004	17 (11)	143 (89)	0.325	19 (12)	141 (88)	0.873
	I	4 (1)	375 (99)		7 (2)	372 (98)		4 (6)	59 (94)		7 (11)	56 (89)	

(F)

		Adrenal Gland Metastasis											
		All Cases						Recurrence Cases					
		Initial Recurrence			Final Recurrence			Initial Recurrence			Final Recurrence		

Factors		Present (%)	Absent (%)	*p*	Present (%)	Absent (%)	*p*	Present (%)	Absent (%)	*p*	Present (%)	Absent (%)	*p*
Sex	male	8 (2)	400 (98)	0.770	12 (3)	396 (97)	0.455	8 (6)	137 (94)	0.999	12 (8)	133 (92)	0.793
	female	4 (1)	276 (99)		5 (2)	275 (98)		4 (5)	74 (95)		5 (6)	73 (94)	
Age	≥70	5 (1)	377 (99)	0.387	7 (2)	375 (98)	0.323	5 (5)	105 (95)	0.768	7 (6)	103 (94)	0.616
	<70	7 (2)	299 (98)		10 (3)	296 (97)		7 (6)	106 (94)		10 (9)	103 (91)	
Smoking	ever	9 (2)	473 (98)	0.999	11 (2)	471 (98)	0.601	9 (5)	155 (95)	0.999	11 (7)	153 (93)	0.398
	never	3 (1)	203 (99)		6 (3)	200 (97)		3 (5)	56 (95)		6 (10)	53 (90)	
Pathology	AD	8 (2)	462 (98)	0.999	13 (3)	457 (97)	0.602	8 (5)	146 (95)	0.999	13 (8)	141 (92)	0.594
	others	4 (2)	214 (98)		4 (2)	214 (98)		4 (6)	65 (94)		4 (6)	65 (94)	
Lymphatic permeation	present	5 (2)	302 (98)	0.999	7 (2)	300 (98)	0.811	5 (4)	115 (96)	0.554	7 (6)	113 (94)	0.318
	absent	7 (2)	374 (98)		10 (3)	371 (97)		7 (7)	96 (93)		10 (10)	93 (90)	
Vascular invasion	present	12 (3)	374 (97)	0.002	17 (4)	369 (96)	0.001	12 (7)	163 (93)	0.074	17 (10)	158 (90)	0.027
	absent	0 (0)	302 (100)		0 (0)	302 (100)		0 (0)	48 (100)		0 (0)	48 (100)	
Pleural invasion	present	7 (5)	136 (95)	0.005	10 (7)	133 (93)	0.001	7 (5)	123 (95)	0.999	10 (8)	120 (92)	0.999
	absent	5 (1)	540 (99)		7 (1)	538 (99)		5 (5)	88 (95)		7 (8)	86 (92)	
Pulmonary metastasis	present	1 (2)	40 (98)	0.525	1 (2)	40 (98)	0.999	1 (6)	17 (94)	0.999	1 (6)	17 (94)	0.999
	absent	11 (2)	636 (98)		16 (2)	631 (98)		11 (5)	194 (95)		16 (8)	189 (92)	
Invasive size	>3 cm	8 (3)	271 (97)	0.078	11 (4)	268 (96)	0.047	8 (6)	130 (94)	0.999	11 (8)	127 (92)	0.999
	≤3 cm	4 (1)	405 (99)		6 (1)	403 (99)		4 (5)	81 (95)		6 (7)	79 (93)	
Lymph node metastasis	present	7 (4)	185 (96)	0.044	9 (5)	183 (95)	0.028	7 (6)	109 (94)	0.770	9 (8)	107 (92)	0.999
	absent	5 (1)	491 (99)		8 (2)	488 (98)		5 (5)	102 (95)		8 (7)	99 (93)	
Adjuvant therapy	performed	7 (4)	163 (97)	0.013	9 (5)	161 (95)	0.018	7 (8)	81 (92)	0.226	9 (10)	79 (90)	0.303
	undone	5 (1)	513 (99)		8 (2)	510 (98)		5 (4)	130 (96)		8 (6)	127 (94)	
Pathological stage	II–III	9 (3)	300 (97)	0.042	12 (4)	297 (96)	0.046	9 (6)	151 (94)	0.999	12 (8)	148 (93)	0.999
	I	3 (1)	376 (99)		5 (1)	374 (99)		3 (5)	60 (95)		5 (8)	58 (92)	

AD, adenocarcinoma; HR, Hazard Ratio; 95% CI, 95% confidence interval.

Table 2. Univariate analysis of time to recurrence and survival after recurrence according to the clinicopathological factors.

			Univariate Analysis					
			Time to Recurrence			Survival after Recurrence		
Factors		*n*	Median Months (Range)	HR (95% CI)	*p*	Median Months (Range)	HR (95% CI)	*p*
Sex	male	145	12 (1–107)	1.32 (1.00–1.75)	0.051	17 (0182)	1.35 (1.04–2.22)	0.038
	female	78	19 (1–119)	1		23.5 (0–121)	1	
Age	≥70	110	12 (1–95)	1.03 (0.79–1.34)	0.841	13 (0–83)	2.03 (1.43–2.89)	0.001
	<70	113	12 (1–119)	1		25 (0–182)	1	
Smoking	ever	164	12 (1–119)	1.06 (0.78–1.43)	0.720	17 (0–180)	1.35 (0.91–1.99)	0.139
	never	59	12 (1–80)	1		24 (1–182)	1	
Pathology	AD	154	17 (1–119)	0.72 (0.54–0.96)	0.025	23.5 (0–180)	0.47 (0.33–0.67)	0.001
	others	69	10 (1–95)	1		10 (1–182)	1	
Lymphatic permeation	present	120	11 (1–80)	1.41 (1.08–1.85)	0.011	21 (0–182)	1.06 (0.75–1.49)	0.758
	absent	103	17 (1–119)	1		17 (0–115)	1	
Vascular invasion	present	175	11 (1–107)	1.64 (1.19–2.27)	0.003	17 (0–182)	1.58 (1.12–2.46)	0.034
	absent	48	24 (1–119)	1		21 (1–121)	1	
Pleural invasion	present	130	12 (1–107)	1.17 (0.89–1.53)	0.258	21 (0–182)	1.04 (0.74–1.47)	0.829
	absent	93	14 (1–119)	1		14 (1–121)	1	
Pulmonary metastasis	present	18	24 (1–119)	1.77 (1.09–2.88)	0.024	13 (1–127)	1.27 (0.72–2.27)	0.410
	absent	205	7 (1–29)	1		20 (0–182)	1	
Invasive size	>3 cm	138	11 (1–119)	1.46 (1.12–1.92)	0.006	17 (0–182)	1.28 (0.90–1.81	0.165
	≤3 cm	85	19 (1–107)	1		21 (0–121)	1	
Lymph node metastasis	present	116	12 (1–80)	1.19 (0.91–1.55)	0.207	20.5 (0–182)	1.22 (0.87–1.71)	0.250
	absent	107	16 (1–119)	1		19 (1–115)	1	
Adjuvant therapy	performed	88	30 (2–107)	1.04 (0.80–1.37)	0.763	22.5 (0–127)	0.72 (0.51–1.02)	0.068
	undone	135	9 (1–119)	1		17 (0–182)	1	
Pathological stage	II–III	160	11 (1–80)	1.67 (1.24–2.26)	0.001	17 (0–182)	1.35 (0.93–1.98)	0.117
	I	63	23 (3–119)	1		21 (1–115)	1	

AD, adenocarcinoma; HR, Hazard Ratio; 95% CI, 95% confidence interval
